# New Chiral P-N Ligands for the Regio- and Stereoselective Pd-Catalyzed Dimerization of Styrene 

**DOI:** 10.3390/molecules16021804

**Published:** 2011-02-22

**Authors:** Lidia Fanfoni, Angelo Meduri, Ennio Zangrando, Sergio Castillon, Fulvia Felluga, Barbara Milani

**Affiliations:** 1 Dipartimento di Scienze Chimiche e Farmaceutiche, Università di Trieste, Via Licio Giorgieri 1, 34127 Trieste, Italy; E-Mails: lfanfoni@units.it (L.F.); angelo.meduri@phd.units.it (A.M.); ezangrando@units.it (E.Z.); ffelluga@units.it (F.F.); 2 Department de Química Analítica i Química Orgànica, Universitat Rovira i Virgili, C. Marcel.lí Domingo s/n 43007 Tarragona, Spain; E-Mail: sergio.castillon@urv.net (S.C.)

**Keywords:** hybrid phosphorus-nitrogen ligands, palladium, alkene dimerization

## Abstract

Two new chiral, enantiomerically pure, hybrid P-N ligands, namely (2*R*,5*S*)-2-phenyl-3-(2-pyridyl)-1,3-diaza-2-phosphanicyclo[3,3,0]octan-4-one (**1**) and (2*R*,5*S*)-2-phenyl-3-(2-pyridyl)-1,3-diaza-2-phosphanicyclo[3,3,0]octane (**2**), have been synthesized starting from L-proline. The two ligands differ in the presence or not of a carbonyl group in the diazaphosphane ring. Their coordination chemistry towards Pd(II) was studied by reacting them with [Pd(CH_3_)Cl(cod)]. A different behaviour was observed: ligand **2** shows the expected bidentate chelating behaviour leading to the mononuclear Pd-complex, while ligand **1** acts as a terdentate ligand giving a dinuclear species. The corresponding cationic derivatives were obtained from the palladium neutral complexes, both as mono- and dinuclear derivatives, and tested as precatalysts for styrene dimerization, yielding *E*-1,3-diphenyl-1-butene regio- and stereoselectively as the sole product. A detailed analysis of the catalytic behaviour is reported.

## 1. Introduction

The concept of *hemilability* has been coined for ligands possessing a combination of soft and hard donor atoms and compounds combining phosphorus- and nitrogen-donor atoms represent a distinguished family of *hemilable* ligands. In particular, *hemilable* hybrid P-N multidentate ligands are able to stabilize metal ions in a variety of oxidation states and geometries. Indeed, the π-acceptor character of phosphorus atom can stabilize a metal center in a low oxidation state, while the nitrogen σ-donor ability makes the metal more susceptible to oxidative addition reactions, features that can play a crucial role in stabilizing intermediate oxidation states and/or geometries during a catalytic cycle [1]. In addition, the possibility of varying the electronic and steric properties of this kind of ligands allows to obtain potentially multidentate ligands that are able to bind or bridge one or more metal ions affording homo- or hetero-, bi- or polymetallic complexes [1,2].

Late transition metal complexes with chiral P-N ligands have found, and currently find, wide application in asymmetric catalysis [3,4,5,6,7,8,9,10,11]. In the field of ethylene homo- and co-, oligo- and polymerization Ni(II) complexes with a variety of P-N ligands were preferentially used [12,13,14,15,16,17]. Palladium complexes containing P-N ligands were applied as precatalysts for CO/alkene copolymerization; very high CO pressures (up to 320 bar) were required to have catalytic activity [17,18,19,20]. Some Pd(II) complexes with P-N ligands, such as phosphino- phosphinito- and phosphonito-oxazolines, were applied in the CO/ethylene and CO/methyl acrylate copolymerization in the study of the stepwise insertion of the monomer on the precatalyst and resulting in the X-ray characterization of some intermediates [21,22,23].

Chiral diazaphospholidines, more generally diaminophosphines, constitute a family of ligands in which two nitrogen atoms are linked to phosphorus in a phosphorus-alkyl or phosphorus-aryl moiety [24]. Recently, a particular attention has been devoted to diazaphospholidine ligands derived from proline, which have been successfully used in different catalytic processes such as asymmetric copper-catalyzed cyclopropanation [4], iridium catalyzed enantio- and regioselective allylic etherification [7], palladium catalyzed enantioselective allylic amination [1], rhodium-catalyzed asymmetric hydroformylation [25].

Alkene di- and oligomerization are reactions of high industrial importance for the synthesis of α-olefins. When considering styrene as the substrate, the selective dimerization to *E*-1,3-diphenyl-1-butene has been studied using different catalytic systems based on palladium salts modified with monophosphines [26], or with “phosphine-free” systems in ionic liquids [27], or, more recently, by applying both ruthenium [28] and iron [29] catalytic systems. The asymmetric codimerization of styrene with ethylene has been also extensively investigated, using nickel, palladium and cobalt complexes with chiral mono and bidentate phosphines [30,31].

**Figure 1:**
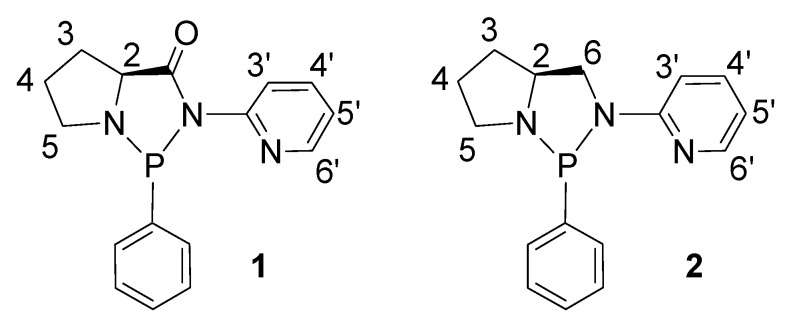
The ligands studied and their numbering scheme.

We have now studied the coordination chemistry towards palladium of a new type of chiral, enantiomerically pure, diazaphospholidine ligands **1**, **2** (Figure 1) derived from proline, together with the catalytic behavior of the relevant Pd-complexes in styrene dimerization. The main structural features of these ligands are: a) the pyridine moiety is linked to the nitrogen atom instead of phosphorus; and b) the electronic properties are modulated for the presence of an amido (compound **1**) or amino group (compound **2**).

## 2. Results and Discussion

### 2.1. Synthesis and Characterization of P-N Ligands ***1*** and ***2***

**Scheme 1:**
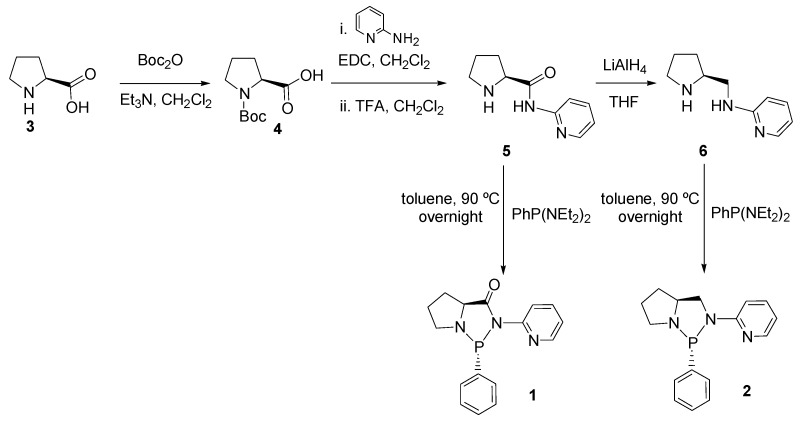
Synthetic pathway for ligands **1** and **2**.

The optically pure ligands **1 **and **2 **were synthesized from amide (*S*)-**5** and amine (*S*)-**6**, respectively, which in turn were prepared from proline. Thus, proline was initially protected to give the Boc-derivative **4** [32], which was reacted with 2-aminopyridine using EDC as condensation reagent (EDC = N-(3-dimethylaminopropyl)-*N*’-ethylcarbodiimide hydrochloride) to provide the amide [33,34], which, after Boc removal in acidic medium [35], afforded **5**. Reduction of **5 **with LiAlH_4_ provided derivative **6** (Scheme 1) [36]. Afterwards, the reaction of **5, 6** with PhP(NEt_2_)_2_ in toluene at 90 °C overnight afforded diaminophosphines **1, 2** in 75% and 80% yield, respectively [11]. In agreement with the literature data [7,37], both ligands were stereoselectively obtained as the single β diastereoisomer, as determined by an NOE experiment performed upon irradiation of H^2^, which resulted in the enhancement of the intensity of the resonance of phenyl protons in the *ortho* position. Since the absolute configuration of C2 is known to be *S*, the stereogenic phosphorus center must have the *R* configuration.

Ligands **1** and **2** were fully characterized in solution by multinuclear NMR spectroscopy (Table 1) and circular dicroism.

### 2.2. Study of Palladium Coordination Chemistry

**Scheme 2:**
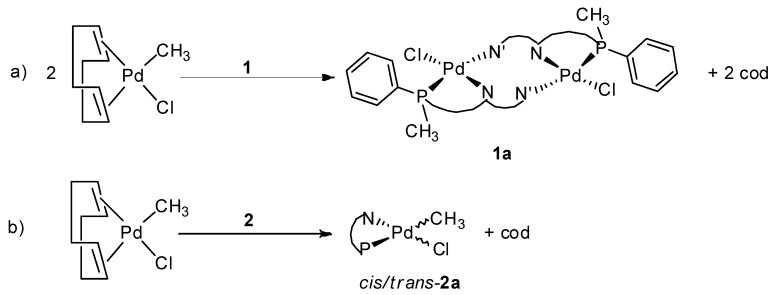
Schematic representation of the reactivity of ligands **1 **(a) and **2 **(b) with [Pd(CH_3_)Cl(cod)].

The coordination chemistry of **1** and **2** to palladium was initially studied by *in situ* NMR spectroscopy by reacting CD_2_Cl_2_ solutions of the ligands with equimolar amounts of [Pd(CH_3_)Cl(cod)] (cod = 1,5-cyclooctadiene). The reaction progress, monitored by ^1^H- and ^31^P-NMR spectroscopy, showed an almost instantaneous coordination of the two ligands to the metal centre: no signal of the free P-N ligand was present after 5 min from the mixing of the two species. On this basis, complexes **1a**, **2a** were synthesized by reacting the Pd-precursor with the P-N ligand in dichloromethane at room temperature and isolated as yellow-orange solids upon addition of *n*-hexane (Scheme 2).

The neutral complexes were fully characterized in CD_2_Cl_2_ solution by multinuclear NMR spectroscopy (Table 1).

**Table 1:** Selected chemical shift values for the ligands and the relevant palladium complexes.^[a]^

P-N/ complexes	^31^P NMR	CIS^[b]^ (δ_l_-δ_c_)	CH_3_^1^H NMR	*J*_P-CH3_ (Hz)
**1**	106.3	-	-	-
**1a**	67.0 (M), 75.6 (m)	39.3 (M), 30.7 (m)	2.12 (M), 2.45 (m)	13.0 (M), 11.5 (m)
**1b**	50.7	55.6	2.38	11.5
**1c**	50.7	55.6	2.36	11.5
**1d**	50.7	55.6	2.38	11.5
**2**	99.7	-	-	-
**2a**	119.9 (m), 133.8 (M)	-20.2 (m), -34.0 (M)	0.69 (M), 1.65 (m)	2.0 (M), 8.5 (m)
**2b^c^**	120.5 (M), 122.9, 134.2	-20.8 (M), -23.2, -34.5	0.58,^d^ 1.61 (M), 1.71	8.0 (M), 7.8
**2c**	122.2 (m), 135.5 (M)	-22.5 (m), -35.8 (M)	0.58 (M), 1.77 (m)	1.8 (M), 8.6 (m)

^[a]^Spectra recorded in CD_2_Cl_2_ at r.t., values in ppm. M = major species, m = minor species; ^[b]^CIS = Coordination Induced Shift for ^31^P in the free ligand (δ_l_) and in the complex (δ_c_); ^[c]^Spectra recorded in CD_2_Cl_2_ at 223 K; ^[d]^The ^1^H-^31^P coupling constant was too small to be measured; the coupling is evident from the ^1^H,^31^P-HMBC experiment.

The NMR analysis showed a clear shift of the characteristic signals of the ligand protons with respect to those of the free P-N, that was diagnostic of the coordination to the palladium centre. In particular, in the ^1^H-NMR spectra of both neutral species **1a**, **2a** the methyl group bound to Pd in the metal precursor generated two doublets at very different chemical shifts: 2.12 e 2.45 ppm for **1a** and 0.69 and 1.65 ppm for **2a **(Table 1). These signals indicated for both complexes the presence, in solution, of two different species in a ratio of 9:1 and 2:1 for **1a** and **2a**, respectively. This evidence was confirmed by the ^31^P-NMR spectra in which two singlets for each complex were recognized. Interestingly, depending on the nature of the P-N ligand, the ^31^P-NMR signals of the complexes had an opposite variation of chemical shift with respect to the same signal in the free ligands: an upfield shift for **1a **(CIS 39.3 and 30.7 ppm) and a downfield shift for **2a** (CIS −34.0 and −20.2 ppm) (Table 1).

**Figure 2:**
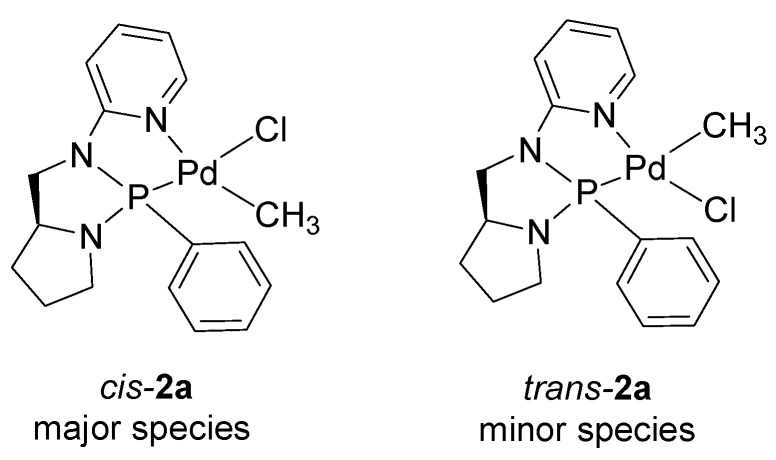
The *cis* and *trans* isomers of complex **2a**.

The comparison of ^1^H- and ^31^P-NMR spectra of **1a** and **2a **suggests that from the reaction of ligands **1** and **2 **with [Pd(CH_3_)Cl(cod)] two different complexes were obtained depending on the nature of the ligand. In particular, for ligand **2 **the data indicated that the isolated species was the expected mononuclear complex [Pd(CH_3_)Cl(**2**)] (**2a**) [17,38]. It is reasonable to assume that the two species observed in solution for **2a** were the *cis* and *trans* isomers differing for the relative position of the Pd-CH_3_ fragment with respect to the two halves of the ligand. Conventionally, we labeled *trans* the isomer having the Pd-CH_3_ in *trans* to the Pd-P bond (Figure 2).

The downfield shifted^ 31^P signals for complex **2a** and the *J*_P-CH3_ values suggested that the major species was the *cis* isomer. This was in agreement with the complex electronic pattern: chlorido was *trans* to the better donor atom (phosphorus), while methyl was *trans* to the poorer donor (nitrogen) (Figure 2) [38] and the groups having the highest *trans* influence were *cis* to each other. In agreement with this signal to proton assignment, the doublet of the methyl group in the *cis* isomer was remarkably upfield shifted with respect to the same signal in the *trans* isomer indicating that it fell into the shielding cone of the phenyl ring bound to phosphorus. The nature of the neutral complex **1a** was elucidated after the characterization of the corresponding cationic derivative.

**Scheme 3:**
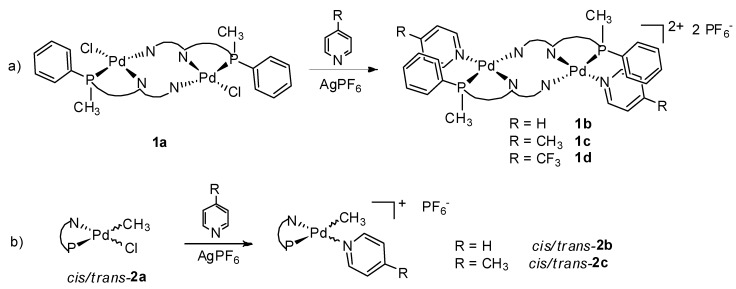
Synthetic pathway for the cationic complexes with ligand **1** (a) and ligand **2** (b).

When the neutral complexes **1a**, **2a** were reacted with AgPF_6_ in the presence of acetonitrile, with the aim to obtain the corresponding monocationic derivatives, no clear product was isolated from the synthetic mixture. It was possible to obtain the cationic species via halogen abstraction on the neutral derivatives by using coordinating ligands stronger than acetonitrile like pyridine, 4-methylpyridine and 4-trifluoromethylpyridine (Scheme 3).

**Figure 3:**
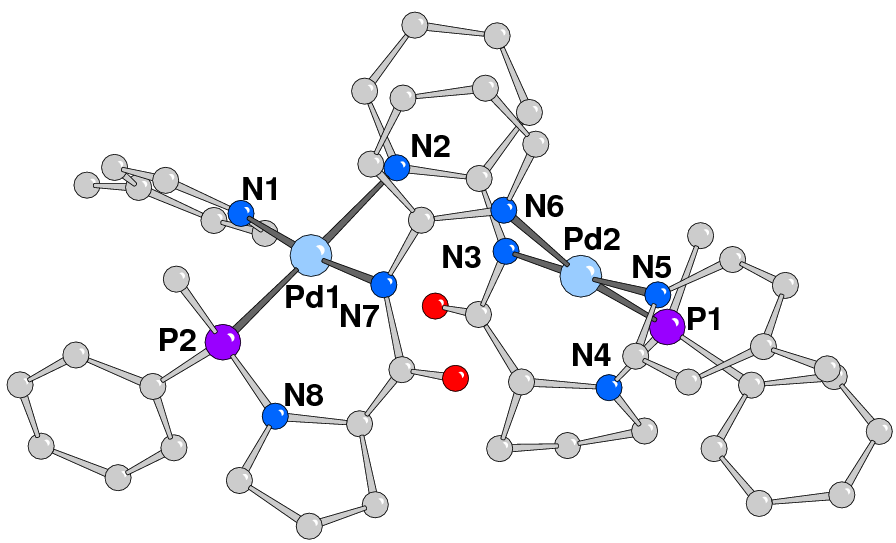
A perspective view of the dinuclear complex 1c.

Even though the quality of the crystals was not excellent, an X-ray single crystal diffraction analysis of complex **1c** was of great usefulness to characterize the palladium coordination sphere and the ligand molecule (Figure 3).

In fact the structural solution (final R value = 15%) provided evidence for the dinuclear nature of this complex (Figure 3; Table 1S) having an approximately *C_2_* symmetry with the *pseudo* twofold axis passing in between the metals. The two ligand molecules acted as a tridentate unit bridging the Pd ions in a head-tail arrangement. Each of these chelated one Pd ion with the phosphorus atom and the amidic nitrogen, that was prone to the coordination due to the cleavage of the P-N_amidic_ bond, and coordinated the other Pd through the nitrogen atom of the pyridine ring. The metal complex geometry was, as usual, square planar with no particular distortions and the palladium ions completed the square planar coordination through the N donor of 4-methylpyridine. The amidic nitrogen was *sp^2^* hybridized as confirmed by the sum of bond angles around N3 and N7 of ca. 358°. The intermetallic separation was 4.638(4) Å and the mean coordination planes formed a dihedral angle of 71.3(4)°.

It was worth noting the presence of π-stacking interactions, occurring between the pyridine rings N2/N6 (distance between centroids of 3.67 Å) and also between each picoline ring and the adjacent phenyl attached to phosphorus (distance of 3.64 and 4.23 Å, respectively for N5 and N1), which stabilized the overall structure.

A peculiarity of this system was the cleavage of the N-P-N heterocycle and the transmethylation reaction occurring between the metal center and the phosphorus atom. This reaction might be seen as a nucleophilic attack of the methyl group, bonded to palladium, at the coordinated phosphine with the opening of the N-P-N heterocycle and the contemporary coordination of the amidic nitrogen to the metal centre. Transmethylation reaction was reported to occur on the complex *trans*-[Pd(CH_3_)I(PPh_3_)_2_] resulting in the exchange of the methyl group on palladium with one phenyl ring on the phosphorus to give [Pd(Ph)I(PPh_3_)(PPh_2_CH_3_)] as product [39]. The reaction here observed might be considered as another example of this type of reactivity, and in this case it occurs on a chiral phosphine.

All cationic complexes were fully characterized by ^1^H- and ^31^P-NMR spectroscopy (Table 1). For complexes **1b-d**, both the ^31^P and the^ 1^H signals were shifted compared to the same signals in the neutral compound **1a**. The signals of the protons of the pyridine-type ligand coordinated to palladium were also present. It was worth noting that the ^31^P and the CH_3_ group signals were not affected by the nature of the fourth ligand, as well as the value of the P-CH_3_ coupling constant, the latter being very similar to the value found for complex **1a **(Table 1). On the basis of these NMR data it was reasonable to assume that all the synthesized complexes containing ligand **1** were dinuclear species with a geometry analogous to that observed in the solid state for **1c**. A dinuclear molecule was also postulated for the neutral complex **1a**. In this case the two species present in solution cannot be the *cis* and *trans* isomers observed for **2a**. A reasonable hypothesis for their nature is that they might be the two different diastereoisomers differing in the absolute configuration of phosphorus.

**Figure 4:**
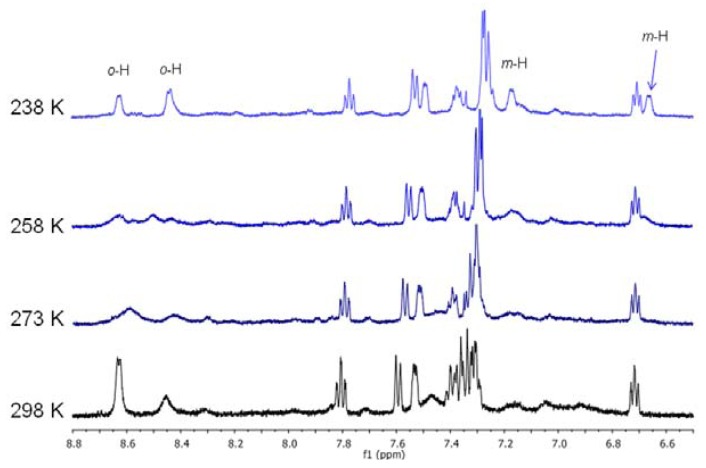
^1^H**-**NMR spectra of **1c**, in CD_2_Cl_2_: variation with temperature (the *ortho* and *meta* protons of 4-picoline are evidenced).

The ^1^H-NMR spectrum of **1c** at room temperature showed a single broad signal at low field for the protons in *ortho* position of 4-picoline, that, in the homonuclear COSY spectrum, was not associated to any cross peak. As the temperature decreased, it became broader, reaching coalescence at 258 K. A further decrease of temperature, up to 238 K, resulted in the appearance of four new peaks at 8.63, 8.44, 7.33 and 6.67 ppm, correlated one to each other by a cross peak in the homonuclear COSY spectrum (Figure 4; Figure 1S). These signals were assigned to the *ortho* and *meta* protons of 4-picoline bound to palladium. In addition to them, even the resonances of the phenyl ring bound to phosphorus varied with temperature. These NMR data indicated the presence of a dynamic process in solution, which mainly involved the 4-picoline, that, due to coordination to palladium, had lost its *C_2_* symmetry axis passing through the nitrogen atom and the *p*-CH_3 _group. Thus, the two halves of 4-picoline were no longer equivalent. This suggested that the fluxional process was a hindered rotation around the Pd-N bond, due to the π-stacking interaction between 4-picoline and the phenyl ring on the phosphorus atom. Indeed, both for *ortho* and *meta* picoline protons, the signal at higher fields could be attributed to the half of the 4-picoline falling into the shielding cone of the phenyl ring.

**Figure 5:**
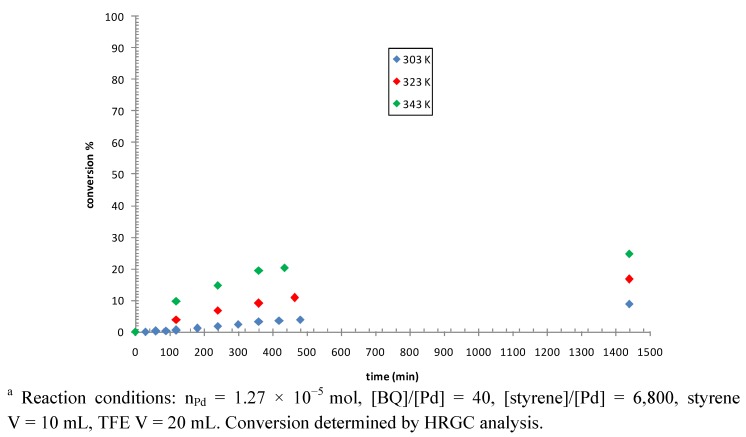
Styrene dimerization: effect of temperature. Precatalyst: **1c**.^a^

**Figure 6:**
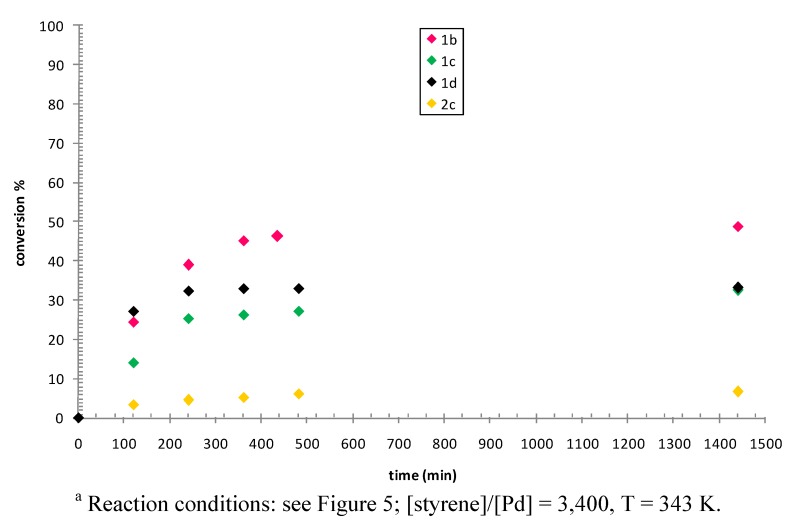
Styrene dimerization: effect of the precatalyst.^a^

Analogous spectra variations with temperature were observed for the cationic derivatives **1b** and **1d** indicating that the same fluxional process occurred regardless to the nature of the pyridine ligand (Figures 2S-5S). As expected, complexes **1b-d** were optically active and their CD spectra (Figure 6S) showed the same Cotton effect, allowing to assign to all of them the same absolute configuration.

**Figure 7:**
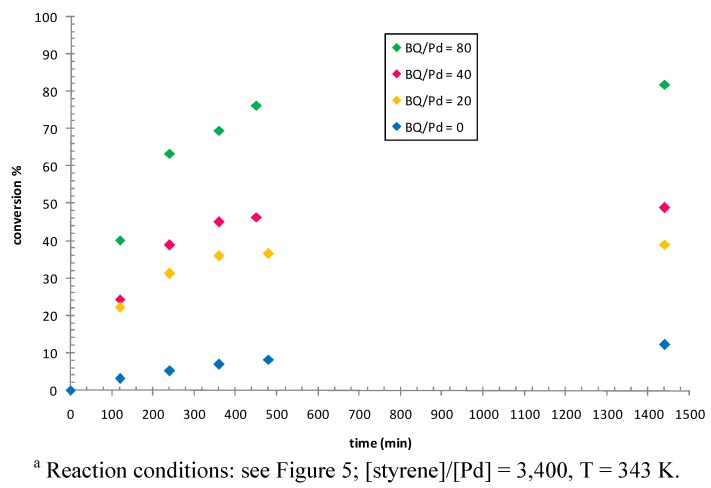
Styrene dimerization: effect of [BQ]/[Pd]. Precatalyst: **1b**.^a^

Complexes **2b-c** were also characterized in CD_2_Cl_2_ solution by multinuclear NMR spectroscopy. In analogy with the neutral compound **2a**, even for the monocationic derivative **2c **the number of signals and their integration in the ^1^H-NMR spectrum indicated the presence of two species in solution that were recognized as the *cis* and *trans* isomers. For complex **2c** this was also confirmed by the two signals in the ^31^P-NMR spectrum. For complex **2b** in the ^1^H-NMR spectrum recorded at room temperature the doublets of the Pd-CH_3_ fragment had chemical shifts values very close to those of the neutral compound **2a**, while all the other signals, including those of pyridine, were quite broad. In the ^31^P-NMR spectrum at room temperature two broad peaks were present too (Figure 7S). Upon decreasing temperature remarkable variations were observed and the decoalescence was reached at 223 K. In particular, in the ^1^H-NMR spectrum three new signals at 8.86, 6.88 and 6.67 ppm appeared (Figure 8S) and they were assigned to the pyridine ring of the P-N ligand; as well as three new doublets at 1.71, 1.61 and 0.58 ppm assigned to the Pd-CH_3_ fragment (Figure 9S). Even in the corresponding ^31^P-NMR spectrum three singlets were observed, which correlated to the doublets of the Pd-CH_3_ group in the ^1^H,^31^P-HMBC spectrum (Figure 10S). On the basis of these NMR data we speculate that **2b** is a mixture of three species which might be assigned as the *cis* and *trans* isomers of [Pd(CH_3_)(py)(**2**)][PF_6_] and the neutral derivative [Pd(CH_3_)Cl(**2'**)(py)] having coordinated to Pd the pyridine in place of the nitrogen of the pyridine ring of the P-N ligand that acted as a monodentate molecule **2'**.

### 2.3. Catalytic Activity of Cationic Complexes 1b-d, 2b-c

**Scheme 4:**
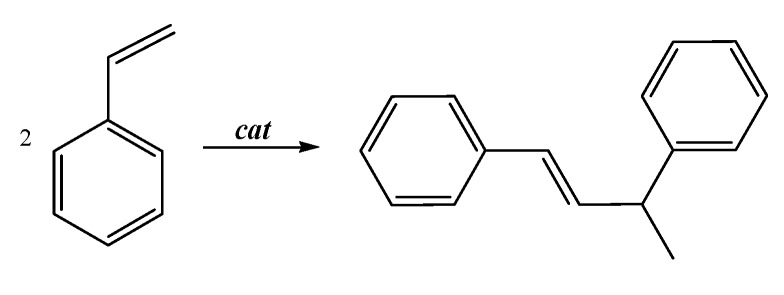
Styrene dimerization reaction.

The cationic complexes **1b-d** were tested as precatalysts for CO/styrene copolymerization under standard conditions: T = 303 K, 1 atm of CO, [styrene]/[Pd] = 6,800, an excess of 1,4-benzoquinone (BQ) with respect to palladium ([BQ]/[Pd] = 40), 24 h, in 2,2,2-trifluoroethanol (TFE). No polymer was isolated at the end of the catalytic runs. After drying the reaction mixture an oil was obtained that was characterized as *E*-1,3-diphenyl-1-butene (Scheme 4). Despite the optical activity of the complexes, the product was obtained as a racemic mixture.

Carbon monoxide was not required for the reaction. The effect of some parameters was studied by using complex **1c** as precatalyst. Increasing the temperature from 303 to 343 K resulted in a remarkable increase of catalytic activity and at 343 K the induction time, observed at 303 and 323 K, was no longer observed (Figure 5, Table 2S). At 343 K 20% conversion was reached after 7 h of reaction and it increased up to 25% after 24 h, indicating catalyst deactivation due to decomposition to palladium black.

A small enhancement in the conversion, up to 32%, was realized by decreasing the styrene to palladium ratio to 3,400 (Figure 11S). By performing the catalytic reactions at T = 343 K and with [styrene]/[Pd] = 3,400 the effect of the nature of the P-N and of the pyridine-type ligand was investigated (Figure 6).

All tested precatalysts generated an active species for styrene dimerization; the maximum conversion reached was 49% with precatalyst **1b**. In all cases the reaction was regiospecific and stereospecific in leading to the *E*-1,3-diphenyl-1-butene as the sole product. The catalytic activity was affected by the nature of the P-N ligand, being all complexes with ligand **1** more active than the complex with ligand **2**. For complexes with ligand **1** the nature of the pyridine-type ligand remarkably affected catalyst activity and stability. Within the first two hours of reaction the trend of the activity might be related to the Lewis basicity of the pyridine-type ligand: it increased on decreasing the Lewis basicity of the ligand, being complex **1d** with the 4-CF_3_-pyridine the most active and **1c** with the 4-CH_3_-pyridine the least active among the three (Figure 6). On prolonging the reaction time, catalyst stability became predominant and, while catalysts generated by complexes **1d** and **1c** deactivated after 4 h, catalyst obtained from **1b** was active for at least 8 h.

In palladium catalyzed oligo- and polymerization reactions the catalyst stability is related to the presence of 1,4-benzoquinone, thus its effect was investigated in this reaction, too (Figure 7, Table 3S). Varying the [BQ]/[Pd] ratio from 0 to 80 resulted in a remarkable increase of the reaction rate, while only a slight effect on catalyst lifetime was observed. This was particularly evident from the values of conversion, measured after 2 h, for the reaction carried out with no addition of BQ and that with [BQ]/[Pd] = 20, of 3.1 and 22.2, respectively. A similar trend was recently observed for the Pd-catalyzed CO/styrene oligomerization reaction: no product was isolated when benzoquinone was not added to the reaction mixture, while a productivity of 185 g PK/g Pd (g PK/g Pd = grams of oligomer per gram of palladium) was obtained performing the oligomerization at [BQ]/[Pd] = 40; however the lack of CO uptake data did not allow to assign this effect to an increase of catalyst lifetime or catalyst activity [40].

These trends differed from the effect that benzoquinone typically shows in CO/alkene co- and ter-polymerization reactions. For the latter reactions a linear relationship between [BQ]/[Pd] ratio and catalyst lifetime was observed together with no effect on the reaction rate [19,41]. The role played by benzoquinone in the copolymerization reactions is well understood and it consists in the oxidation of Pd(0) to Pd(II) with the concomitant formation of hydroquinone [42,43,44,45]. The Pd species resulting from this reaction was a Pd-alkoxy derivative and the corresponding carbo-alkoxy group was found as one end group of the polymeric chains. In the currently investigated reaction, as well as in the CO/styrene oligomerization, no alkoxy (or carbo-alkoxy) group was incorporated into the reaction product. In addition, in styrene dimerization benzoquinone affected catalyst activity rather than catalyst lifetime, thus indicating that it increased the number of active sites. The available experimental data do not allow to make any speculation on the specific activation reaction involving benzoquinone.

Complex **1b** was tested as precatalyst for styrene/ethylene codimerization under mild reaction conditions (1.5 bar of ethylene, T = 343 K, [styrene]/[Pd] = 3,400, [BQ]/[Pd] = 40). No codimer was formed, being *E*-1,3-phenyl-1-butene the unique reaction product detected, thus indicating the selective reactivity of this complex toward the vinyl arene with respect to the aliphatic alkene.

**Scheme 5:**
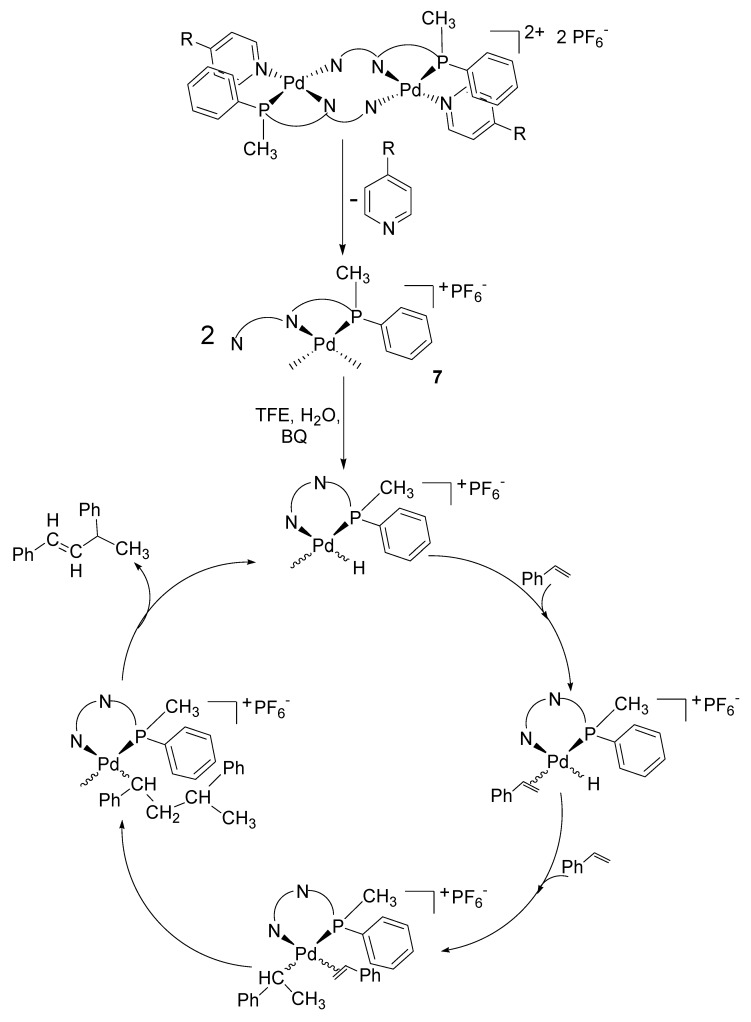
Proposed catalytic cycle.

Despite the fact that the collected experimental data are very preliminary, the following hypothesis for the mechanism of the dimerization reaction is proposed (Scheme 5). The trend of activity with respect to the nature of the pyridine-type ligand indicated that the dissociation of this ligand was required for the catalysis and it might be followed by the cleavage of the Pd-N bond of the pyridine ring of the P-N ligand creating, in this way, a mononuclear intermediate **7** with two *cis* coordination sites available for the catalysis (Scheme 5). The nature of the catalytic product indicated that the catalyst was a Pd-hydride species, that might be formed by the reaction of **7** with trifluoroethanol and/or water (present in traces in the solvent). This reaction might involve benzoquinone and a rearrangement of the ligand: the amidic nitrogen might undergo an electrophilic attack with the cleavage of the related Pd-N bond followed by the coordination of the pyridine ring of the P-N ligand yielding a Pd-H monocationic species, where styrene coordination and insertion took place leading to the Pd-alkyl intermediate. On the latter the second molecule of the vinyl arene coordinated and inserted followed by β-hydrogen elimination, which led to *E*-1,3-phenyl-1-butene and to the Pd-H that might reenter the catalytic cycle or decompose to Pd metal (Scheme 5). Both styrene migratory insertions were regiospecific and occurred with secondary regiochemistry.

## 3. Experimental

### 3.1. General

^1^H- and ^13^C-NMR spectra were run on a JEOL EX 400 Spectrometer at 400 MHz and on a Varian 500 spectrometer at 500 MHz for proton and at 100.1 and 125.68 MHz for carbon, respectively, by using deuterochloroform and deuterodichloromethane as the solvents and referenced to the solvent residual peak to TMS (CDCl_3_ at 7.26 ppm, CD_2_Cl_2_ at 5.32 ppm for proton; CDCl_3_ at 77.0 ppm and CD_2_Cl_2_ at 54.00 ppm for carbon), at 25 °C where not differently indicated. ^31^P-NMR spectra were recorded at 202.32 MHz on a Varian 500 spectrometer and automatically referenced to H_3_PO_4_. NOESY experiments were performed according to the automatic parameters of the software with a mixing time of 500 ms. Optical rotations and CD spectra were determined at 25 °C. TLC were performed on silica gel, using light petroleum - ethyl acetate or dichloromethane - methanol mixtures as the eluent. Flash chromatography was run on silica gel, 230–400 mesh using mixtures of light petroleum 40–70 °C or chloroform and ethyl acetate as the eluent. HRGC analysis were run on a SE 30 capillary column (30 m × 0.32 mm) carrier gas He 50 KPa, split 1:60. The chiral P-N ligands and the related complexes were synthesized under argon atmosphere with Schlenk technique and using freshly distilled and degassed solvents.

### 3.2. Synthesis of P-N Ligands

*(S)-(-)-N-(pyridine-2-yl)pyrrolidine-2-carboxamide* ((*S*)-**5**). Compound (*S*)-**4 **(1.000 g, 4.65 mmol) was dissolved in dichloromethane (20 mL). The solution was cooled to 0 °C, and EDC (0.908 g, 4.74 mmol) was added. The solution was stirred at 0 °C for 0.5 h and then 2-aminopyridine (0.439 g, 4.65 mmol) was added in one portion. The mixture was left at room temperature and followed by TLC (EtOAc/CH_3_OH/NH_3_ 8:2:0.1). After removal of the solvent under reduced pressure, the residue was purified by column chromatography on silica gel (petroleum ether/ethyl acetate 3:7, petroleum ether/ethyl acetate 4:6, then petroleum ether/ethyl acetate/ammonia 4:6:0.2) to give Boc-(*S*)-**5 **as a white solid. [α]_D_ –71 (*c* 0.25, CH_3_OH); ^1^H-NMR (400 MHz, CDCl_3_): *δ* 1.26–1.42 (bs, 9H, *t*-Bu), 1.69 (bs, 1H), 1.91 (m, 2H), 2.24 (bs, 1H), 3.53 (bs, 2H), 4.32 (bs, 1H, C*H*CO), 7.01 (bs, 1H), 7.68 (bs, 1H), 8.21 (d, *J* = 8.0 Hz, 1H), 8.28 (dm, 1H), 9.22 (bs, 1H, N*H*); ^13^C-NMR (100.1 MHz, CDCl_3_): *δ* 21.4, 24.8, 28.3, 47.2, 62.3, 80.96, 113.8, 119.78, 138.22, 148.0, 151.8, 155.2, 172.0; ESI-MS: 292 (M+H^+^), 314 (M+Na^+^).

*Boc*-(*S*)-**5 **(1.508 g, 5.17 mmol) was dissolved in a trifluoroacetic acid solution (15 mL, 40% in CH_2_Cl_2_) at 0 °C and the solution was stirred for 2 h at room temperature. Then the trifluoroacetic acid was neutralized by addition of Et_3_N (15 mL, 0.10 mmol) at 0 °C. The solution was washed with water, dried over anhydrous Na_2_SO_4_, and concentrated under reduced pressure obtaining (*S*)-**5 **as a yellow oil in 90% yield. [α]_D_ –56.4 (*c* 0.5, CH_3_OH); IR (neat) ν_max_: 3276, 2969, 2870, 1692, 1589, 1574, 1510, 1434, 1299, 1148, 1095, 779 cm^−1^;^ 1^H-NMR (400 MHz, CDCl_3_): *δ* 1.69 (m, 2H), 1.97 (m, 1H), 2.16 (m, 1H), 2.54 (bs, 1H, N*H*-amine), 3.00 (m, 2H), 3.84 (dd, *J* = 4.0, 5.2 Hz, 1H, C*H*CO), 6.97 (ddd, *J* = 0.8, 2.4, 4.8 Hz, 1H), 7.64 (td, *J* = 2.0, 8.0 Hz, 1H), 8.20 (d, *J* = 8.4 Hz, 1H), 8.24 (dm, *J* = 4.8 Hz, 1H), 10.17 (bs, 1H, N*H*-amide); ^13^C-NMR (100.1 MHz, CDCl_3_): *δ* 26.3, 30.9, 47.4, 61.0, 113.6, 119.7, 138.3, 148.0, 151.2, 174.4; ESI-MS: 192 (M+H^+^), 214 (M+Na^+^).

*(S)-(+)-N-(pyrrolidin-2-yl-methyl)pyridin-2-amine* ((S)-**6**). To a cooled (-10 °C) and stirred solution of the amide (*S*)-**5** (0.304 g, 1.05 mmol) in dry THF (42 mL) LiAlH_4_ (0.470 g, 12.4 mmol) was added portionwise, and the mixture was stirred at r.t. until almost all starting material was consumed. The mixture was concentrated and the residue diluted with CH_2_C1_2_. The reaction was quenched by the careful addition of 2 M NaOH by using an ice-bath. Stirring was continued to obtain a clear organic layer and the white residue was filtered off. The two-phase mixture was separated and the aqueous layer was extracted with CH_2_Cl_2_. The combined organic phases were dried over anhydrous Na_2_SO_4_, and evaporated. The product (*S*)-**6** was obtained as a yellow oil in 90% yield and used without further purifications. [α]_D_ +28.9 (*c* 0.35, CH_3_OH); IR (neat) ν_max_: 3287, 2961, 2870, 1606, 1518, 1488, 1291, 771, 736 cm^−1^. ^1^H-NMR (400 MHz, CDCl_3_): *δ* 1.47 (m, 1H), 1.75 (m, 2H), 1.89 (m, 1H), 2.52 (bs, 1H, N*H*-prolinic amine), 2.93 (td, *J* = 0.8, 6.4 Hz, 1H ), 3.14 (m, 1H), 3.39 (m, 2H), 4.94 (bs, 1H, N*H*-amine), 6.4 (dt, *J* = 0.8, 8.4 Hz, 1H), 6.53 (ddd, *J* = 0.8, 2.0, 5.2 Hz, 1H), 7.36 (ddd, *J* = 0.4, 1.6, 6.8 Hz, 1H), 8.04 (dm, *J* = 5.6 Hz, 1H);^ 13^C-NMR (100.1 MHz, CDCl_3_): *δ* 25.5, 29.0, 46.2, 58.3, 107.9, 112.8, 137.3, 147.7, 158.8; ESI-MS: 178 (M+H^+^).

#### General procedure for preparation of P-N ligands **1** and **2**

Amide (*S*)-5 or amine (*S*)-6 (1.05 mmol) was weighted in a Schlenk and azeotropically dried three times with distilled and degassed toluene. 5 mL of toluene were then added, and bis(diethylamino)phenylphosphine (244 μL, 0.94 mmol) was added dropwise under Ar at 0 °C. The reaction mixture was heated to 90 °C and stirred overnight. After cooling, the solution was evaporated *in vacuo*. Compound 1 was washed 7 times with distilled and degassed *n*-pentane, giving a white solid. Compound 2 was used as a crude reaction product (viscous orange oil) without further purifications.

*(2R,5S)-(-)-2-phenyl-3-(2-pyridyl)-1,3-diaza-2-phosphanicyclo[3,3,0]octan-4-one* (1). [α]_D_ −128.9 (*c* 0.35, CH_3_OH); ^1^H-NMR (500 MHz, CD_2_Cl_2_): *δ* 1.72 (m, 1H, C*H_2_*, H^4^), 1.87 (m, 1H, C*H_2_*, H^4^), 2.18 (m, 1H, C*H_2_*, H^3^), 2.24 (m, 1H, C*H_2_*, H^3^), 3.39 (m, 1H, C*H_2_*, H^5^), 3.46 (m, 1H, C*H_2_*, H^5^), 4.16 (dd, *J* = 3.0, 9 Hz, 1H, C*H*, H^2^), 7.04 (dd, *J* = 2.0, 5.5 Hz, 1H, H^4’^), 7.35 (m, 3H, Ph), 7.57 (m, 2H, Ph), 7.73 (td, *J* = 1.5, 8.0 Hz, 1H, H^5’^), 8.25 (dm, *J* = 4.0 Hz, 1H, C*H*, H^3’^), 8.33 (dm, *J* = 8.5 Hz, 1H, C*H*, H^6’^);^ 13^C-NMR (100.1 MHz, CDCl_3_): *δ* 26.5 (C^4^), 30.9 (C^3^), 55.4 (C^5^), 68.3 (C^2^), 114.4 (C^6’^), 120.1 (C^4’^), 128.6 (C_Ph_-P), 129.8 (Ph), 130.0 (Ph), 138.3 (C^5’^), 147.7 (C^3’^), 152.4 (C^2’^), 178.1 (*C*O); ^31^P-NMR (202.32 MHz, CD_2_Cl_2_): *δ* 106.29; ESI-MS: 298.1 (M+H^+^), 320.1 (M+Na^+^).

*(2R,5S)-(-)-2-phenyl-3-(2-pyridyl)-1,3-diaza-2-phosphanicyclo[3,3,0]octane* (**2**). [α]_D_ −170.6 (*c* 0.485, CH_3_OH); ^1^H-NMR (500 MHz, CD_2_Cl_2_): *δ* 1.78 (m, 2H, C*H_2_*, H^4^ and 1H, C*H_2_*, H^3^), 2.07 (m, 1H, C*H_2_*, H^3^), 3.08 (m, 1H, C*H_2_*, H^6^), 3.31 (m, 1H, C*H_2_*, H^5^), 3.37 (m, 1H, C*H_2_*, H^5^), 3.55 (m, 1H, C*H_2_*, H^6^), 4.00 (q, *J* = 2.0, 18.5 Hz, 1H, C*H*, H^2^), 6.61 (d, *J* = 8.0 Hz, 1H, H^5’^), 6.64 (ddd, *J* = 1.0, 2.0, 5.0 Hz, 1H, H^3’^), 7.30 (m, 3H, Ph), 7.47 (m, 2H, Ph and 1H, H^4’^), 8.10 (dm, *J* = 4.5 Hz, 1H, H^6’^); ^13^C-NMR (100.1 MHz, CD_2_Cl_2_): *δ* 26.1 (C^4^), 31.3 (C^3^), 52.2 (C^6^), 52.6 (C^5^), 64.8 (C^2^), 108.5 (C^5’^), 113.7 (C^3’^), 128.5 (Ph), 129.2 (Ph), 129.9 (C_Ph_-P), 137.9 (C^4’^), 148.7 (C^6’^); ^31^P-NMR (202.32 MHz, CD_2_Cl_2_): *δ* 99.71; ESI-MS: 284.1 (M+H^+^).

### 3.3. Synthesis of Pd Complexes

All complexes were prepared starting from [Pd(CH_3_)Cl(cod)], following the procedure reported in the literature. Briefly, ligand **1** or **2** (1.50 mmol) was added to a solution of [Pd(CH_3_)Cl(cod)] (332 mg, 1.25 mmol) in freshly distilled dichloromethane (5 mL) and stirred at room temperature. After 1.5 h the reaction mixture was concentrated and the product precipitated as a yellow - orange solid upon addition of n-hexane.

[Pd(CH_3_-**1**)Cl]_2 _(**1a**): yield 70%; [α]_D_ −7.6 (c 0.29, CH_3_OH); IR (nujol) ν_max_: 1693 cm^−1^; ^1^H-NMR (500 MHz, CD_2_Cl_2_): δ 1.94–2.11 (m, 2H, CH_2_, H^4^ and 1H, CH_2_, H^3^ ), 2.12 (d, J = 13.0 Hz, 3H, CH_3_-P), 2.45 (d, J = 11.5 Hz, 3H, CH_3_-P minor isomer), 2.61 (m, 1H, CH_2_, H^3^), 3.29 (m, J = 7.5 Hz, 1H, CH_2_, H^5^), 3.39 (m, 1H, CH_2_, H^5^), 3.81 (m, 1H, CH, H^2^), 6.91 (t, J = 6.0 Hz, 1H, H^5’^), 7.55 (tm, J = 2.5, 7 Hz, 2H, m-H-Ph), 7.59 (m, 1H, p-H-Ph), 7.68 (td, J = 1.5, 8.0 Hz, 1H, H^4’^), 7.79 (d, J = 8.5 Hz, 1H, H^3’^), 7.96 (m, J = 2.5 Hz, 1H, H^6’^), 8.06 (dd, J = 5.5, 7.5 Hz, 2H, o-H-Ph ); ^13^C-NMR (125 MHz, CD_2_Cl_2_): δ 15.3 (d, J = 182.5 Hz, CH_3_-P), 25.4 (d, J = 48.5 Hz, C^4^), 29.7 (d, J = 38.5 Hz, C^3^), 49.6 (C^5^), 65.0 (C^2^), 115.2 (C^3’^), 117.9 (C^5’^), 128.8 (p-Ph), 129.6 (d, J = 47.5 Hz, o-Ph), 132.3 (d, J = 51 Hz, m-Ph), 133.0 (Ph-P), 140.5 (C^4’^), 144.7 (C^6’^), 170.3 (CO); ^31^P-NMR (202.32 MHz, CD_2_Cl_2_): δ 67.05 (minor isomer), 75.57 (major isomer). Isomeric ratio 9:1.

*[Pd(CH_3_)Cl(**2**)]* (**2a**): yield 66%; [α]_D_ −20.4 (*c* 0.12, CH_3_OH);^ 1^H-NMR (500 MHz, CD_2_Cl_2_): *δ* 0.69 (d, *J* = 2.0 Hz, 3H, C*H_3_*-Pd, *cis* isomer), 1.57 (m, 1H, H^3^, *cis* isomer), 1.65 (d, *J* = 8.8 Hz, 3H, C*H_3_*-Pd, *trans* isomer), 1.94 (m, 2H, H^4^, *cis* isomer), 2.18 (m, 1H, H^3^, *cis* isomer), 2.64–3.00 (m, aliphatic proton of the *trans* isomer), 3.29 (m, 1H, H^6^* cis* isomer), 3.49 (m, 1H, H^5^, *cis* isomer), 3.70 (m, 1H, H^6^ and 1H, H^5^, *cis* isomer), 3.86 (m, aliphatic protons, *trans* isomer), 4.06 (m, 1H, H^2^* cis* and aliphatic protons of the *trans* isomer), 6.67 (d, *J* = 8.5 Hz, 1H, H^3’^), 6.85 (d, *J* = 8.0 Hz, 1H, H^3’^, *trans* isomer), 6.95 (m, 1H, H^5’^ of both *cis* and *trans* isomers), 7.07 (t, *J* = 7.5 Hz, Ph, *trans* isomer), 7.46 (m, 3H, aromatic protons of both *cis* and *trans* isomers), 7.73 (m, 2H, Ph and 1H, H^4’^, *cis* isomer and Ph H^4’^, *trans* isomer), 8.98 (m, 1H, H^6’^); ^13^C-NMR (125 MHz, CD_2_Cl_2_): *δ* 0.69 (d, *J* = 2.0 Hz, 3H, *C*H_3_-Pd, *cis* isomer), 18.5 (*C*H_3_-Pd, *trans* isomer), 26.2 (C^4^, *cis* isomer), 32.8 (C^3^, *cis* isomer), 51.6 (C^6^, *cis* isomer), 52.2 (C^5^, *cis* isomer), 65.9 (C^2^, *cis* isomer), 109.6 (C^3’^, *cis* isomer), 110.4 (C^3’^, *trans* isomer), 116.5 (C^5’^, *cis* isomer), 123.4 (C^5’^, *trans* isomer), 127.0 (aromatic carbon, *trans* isomer), 128.6 (aromatic carbon, *cis* isomer), 130.2 (aromatic carbon, *cis* isomer), 131.5 (aromatic carbon, *trans* isomer), 137.7 (aromatic carbon, *cis* isomer), 139.9 (C^4’^, *cis* isomer and C^4’^, *trans* isomer), 148.7 (C^6’^, *cis* isomer); ^31^P-NMR (202.32 MHz, CD_2_Cl_2_): *δ* 119.9 (*cis* isomer), 133.7 (*trans* isomer); Ratio *cis*/*trans =* 2:1.

### 3.4. Synthesis of the Cationic Complexes ***1b-d***, ***2b-c***

All complexes were obtained starting from the corresponding neutral derivatives upon addition of AgPF_6_ in a mixture of CH_2_Cl_2 _and pyridine or pyridine derivative. In particular, the neutral complex 1a (0.22 mmol) was dissolved in the minimal amount of CH_2_Cl_2_ under argon and kept in the dark. A solution of AgPF_6_ in CH_2_Cl_2_ (0.0615 g, 0.24 mmol) was added, and then dropwise the pyridine derivative leading to the precipitation of AgCl. After 1.5 h, the solution was filtered over Celite and concentrated to minimal volume *in vacuo*. Upon addition of diethyl ether the product precipitated as a white solid.

*[Pd(CH_3_-**1**)(Py)]_2_[PF_6_]_2_* (**1b**): yield 80%, [α] +90.9 (c = 0.11 in CH_3_OH); IR (nujol) ν_max_: 1706, 842, 557 cm^−1^; ^1^H-NMR (500 MHz, CD_2_Cl_2_, 233 K): *δ* 2.09 (m, 2H, C*H_2_*, H^5^), 2.32 (m, 1H, C*H_2_*, H^3^), 2.38 (d, *J* = 11.5 Hz, 3H, C*H_3_*-P), 2.92 (m, 1H, C*H_2_*, H^3^ and 1H, C*H_2_*, H^4^), 3.21 (m, 1H, C*H_2_*, H^4^), 5.03 (m, *J* = 4.5, 11.0 Hz, 1H, C*H*, H^2^), 6.72 (t, *J* = 6.0 Hz, 1H, H^5’^), 6.87 (t, *J* = 6.5 Hz, 1H, *m*-Py), 7.33 (m, 5H, Ph and 1H, *m*-Py), 7.56 (m, 1H, H^6’^ and 1H, *p*-Py), 7.62 (d, *J* = 8.0 Hz, 1H, H^3’^), 7.81 (t, *J* = 7.5 Hz, 1H, H^4’^), 8.66 (d, *J* = 5.5 Hz, 1H, *o*-Py), 8.85 (d, *J* = 5.5 Hz, 1H, *o*-Py); ^31^P-NMR (202.32 MHz, CD_2_Cl_2 _298 K): *δ* 50.73.

*[Pd(CH_3_-**1**)(4-CH_3_Py)]_2_[PF_6_]_2_* ( **1c**): yield 85%, IR (nujol) ν_max_: 1711, 842, 557 cm^−1^;^ 1^H-NMR (500 MHz, CD_2_Cl_2_, 238 K): *δ* 2.09 (m, 2H, C*H_2_*, H^5^), 2.20 (s, 3H, C*H_3_*-Py), 2.29 (m, 1H, C*H_2_*, H^3^), 2.35 (d, *J* = 11.5 Hz, 3H, C*H_3_*-P), 2.89 (m, 1H, C*H_2_*, H^4^), 2.93 (m, 1H, C*H_2_*, H^3^), 3.20 (m, 1H, C*H_2_*, H^4^), 5.00 (m, 1H, C*H*, H^2^), 6.67 (bs, 1H, *m*-4-CH_3_Py), 6.71 (t, *J* = 5.5 Hz, 1H, H^5’^), 7.17 (bs, 1H, *m*-4-CH_3_Py), 7.34 (m, 5H, aromatic proton), 7.53 (m, 1H, H^6’^), 7.60 (d, *J* = 8.0 Hz, 1H, H^3’^), 7.80 (dt, *J* =1.5, 7.5 Hz, 1H, H^4’^), 8.44 (bs, 1H, *o*-4-CH_3_Py), 8.63 (bs, 1H, *o*-4-CH_3_Py); ^31^P-NMR (202.32 MHz, CD_2_Cl_2 _298 K): *δ* 50.73.

*[Pd(CH_3_-**1**)(4-CF_3_Py)]_2_[PF_6_]_2 _*(**1d**): yield 75%, [α] +120.5 (c = 0.22 in CH_3_OH); ^1^H-NMR (500 MHz, CD_2_Cl_2_, 233 K): *δ* 2.13 (m, 2H, C*H_2_*, H^5^), 2.36 (m, 1H, C*H_2_*, H^3^), 2.40 (d, *J* = 11.5 Hz, 3H, C*H_3_*-P), 2.95 (m, 1H, C*H_2_*, H^4^ and 1H, C*H_2_*, H^3^), 3.22 (m, 1H, C*H_2_*, H^4^), 5.06 (m, 1H, C*H*, H^2^), 6.77 (t, *J* = 6.0 Hz, 1H, H^5’^), 7.08 (bs, 1H, *m*-4-CF_3_Py), 7.22 (bs, 1H, *m*-4-CF_3_Py), 7.31 (m, 3H, aromatic protons), 7.40 (m, 2H, aromatic protons) 7.66 (m, 1H, H^6’^ and 1H, H^3’^), 7.85 (t, *J* = 6.5 Hz, 1H, H^4’^), 8.97 (d, *J* = 6.0 Hz, 1H, *o*-4-CF_3_Py), 9.18 (d, *J* = 6.0 Hz, 1H, *o*-4-CF_3_Py); ^31^P -NMR (202.32 MHz, CD_2_Cl_2_ 298 K): *δ* 50.73.

The synthesis of complexes 2b-c was performed following a procedure analogous to that of 1b-d, with the unique difference that AgPF_6_ was dissolved in pyridine (or in 4-picoline) and the solution was added to the reaction mixture.

*[Pd(**2**)(CH_3_)(Py)][PF_6_]* (**2b**): IR (nujol) ν_max_: 838, 555 cm^−1^; ^1^H-NMR (500 MHz, CD_2_Cl_2_,223 K): *δ* 0.58 (3H, C*H_3_*-Pd), 1.57 (b, H^3^), 1.61 (3H, *J* = 8.0 Hz, C*H_3_*-Pd, M isomer), 1.71 (3H, *J* = 7.8 Hz, C*H_3_*-Pd), 2.02 (b, H^3^), 2.52 (b, H^4^), 2.77 (b, H^3^), 3.23 (t, *J* = 10.3 Hz, H^6^), 3.33 (m, H^6^), 3.45 (b), 3.64 (m, H^5^ H^6^), 3.84 (b, H^5^), 3.94 (b, H^6^), 4.01 (b, H^2^), 4.09 (b, H^2^), 6.67 (d, H^3'^), 6.88 (d, H^3'^, M isomer), 6.96 (b, H^5'^), 7.05 (b, aromatic protons of phenyl ring), 7.40-7.44 (b, aromatic protons of phenyl ring and pyridine), 7.54 (b, aromatic protons of phenyl ring and pyridine), 7.72 (b, H^4'^), 7.78 (b, H^4'^, M isomer), 7.84 (b, aromatic protons), 7.89 (b, aromatic protons), 8.60 (b, aromatic protons), 8.86 (b, H^6'^); ^31^P- NMR (202.32 MHz, CD_2_Cl_2_, 223 K): *δ* 120.5, 122.9, 134.2.

*[Pd(**2**)(CH_3_)(4-CH_3_Py)][PF_6_]* (**2c**): yield% [α]_D_ −12.3 (*c* 0.105, CH_3_OH);^ 1^H-NMR (500 MHz, CD_2_Cl_2_): *δ* 0.58 (d, *J* = 1.8 Hz, 3H, C*H_3_*-Pd, *cis* isomer), 1.05 (t, aliphatic protons, *trans* isomer), 1.37 (t, aliphatic protons, *trans* isomer), 1.70 (m, 1H, H^3^, *cis* isomer), 1.77 (d, *J* = 8.6 Hz, 3H, C*H_3_*-Pd, *trans* isomer), 2.07 (m, 2H, H^4^, *cis* isomer), 2.28 (m, 1H, H^3^, *cis* isomer), 2.32 (s, 3H, CH_3_-Py, *trans* isomer), 2.41 (s, 3H, CH_3_-Py, *cis* isomer), 2.77 (m, aliphatic proton, *trans* isomer), 3.07 (m, 1H, H^4^* cis* isomer), 3.38 (m, 1H, H^6^, *cis* isomer), 3.59 (m, 1H, H^5^, *cis* isomer), 3.67 (m, 1H, H^5^, *cis* isomer), 3.77 (m, 1H, H^6^, *cis* isomer), 4.15 (m, 1H, H^2^, *cis* isomer), 6.86 (m, 2H, *p*-Ph, *cis* and *trans* isomer), 7.05 (m, 2H, Ph, *trans* isomer), 7.14 (d, 2H, *m*-CH_3_-Py, *trans* isomer), 7.24 (d, 2H, *m*-CH_3_-Py, *cis* isomer), 7.31 (d, aromatic protons), 7.40 (m, 1H, Ph, *trans* isomer), 7.54 (m, aromatic protons and *o*-Ph, *cis* isomer), 7.74 (m, *m*-Ph, *cis* isomer), 7.83 (t, *m*-Ph, *cis* isomer), 7.88 (m, Ph, *trans* isomer), 8.05 (d, 2H, *o*-CH_3_-Py, *trans* isomer), 8.44 (d, 2H, *o*-CH_3_-Py, *cis* isomer), 8.50 (b, 2H, aromatic protons of *cis* and *trans* isomer); ^31^P-NMR (202.32 MHz, CD_2_Cl_2_): *δ* 122.2 (*trans* isomer), 135.5 (*cis* isomer). Ratio *cis*/*trans* 2:1.

### 3.5. Dimerization Reaction

All catalytic experiments were carried out in a three-necked, thermostated, 75 mL glass reactor equipped with a magnetic stirrer and connected to a temperature controller. After establishment of the reaction temperature, the precatalyst, 1,4-benzoquinone, styrene and TFE were placed inside. The system was stirred at the same temperature for 24 h. At the end of the reaction time the two layers formed were separated (dimer being the lower density phase), the Pd(0) residue was filtered over Celite and washed with dichloromethane. The product was dried under *vacuo*. IR (neat) ν_max_: 3081, 3059, 3025, 2964, 2928, 2870, 1945, 1873, 1806, 1599, 1492, 1450, 965 cm^−1^;^ 1^H-NMR (400 MHz, CDCl_3_): *δ* 1.47 (d, 3H, *J* = 7.0 Hz, C*H_3_*CH), 3.65 (m, 1H, CH_3_C*H*), 6.42 (m, 2H, *J* = 5.2, 16.2 Hz, *H*C=C*H trans*), 7.19-7.48 (m, 10H, Ph); ^13^C-NMR (125 MHz, CDCl_3_): *δ* 21.2, 42.5, 126.1, 126.2, 127.0, 127.3, 128.5, 135.1, 137.5, 145.6.

### 3.6. Codimerization Reaction

A 50 mL Büchi Tinyclave glass reactor was used for this reaction. The Tinyclave was charged with 1,4-benzoquinone, **1b** (1.3·× 10^−5^ mol), styrene (10 mL), TFE (20 mL), connected to the ethylene tank and pressurized at the desired pressure. The reactor was placed in an oil bath and warmed up to 343 K. The mixture was stirred for 24 h. The reactor was vented, the solution was filtered over Celite to remove palladium black and evaporated under vacuum obtaining a yellow oil.

## 4. Conclusions

In this work we report the synthesis of two new, chiral, enantiomerically pure, hybrid P-N ligands, together with the study of their coordination chemistry towards palladium and the catalytic behavior of the synthesized complexes in styrene dimerization reactions. Starting from the same Pd precursor, complexes of different nature were obtained depending on the nature of the ligand. In particular, ligand **1** having an amidic nitrogen led to a Pd-dinuclear species with the concomitant transfer of the methyl group from the metal centre to the phosphorus atom, while ligand **2** gave the expected mononuclear derivative. This different behavior might be related to the different nature of the nitrogen atom deriving from 2-aminopyridine: in ligand **1** this nitrogen atom is *sp^2^* hybridized enforcing a planar, more strained, conformation of the N-P-N heterocycle, while in ligand **2** this nitrogen atom is *sp^3^* hybridized and thus the related heterocycle should result more flexible and, as a consequence, more stable than in ligand **1**.

The monocationic palladium complexes were found to generate catalysts with modest activity for styrene dimerization reaction leading, regio- and stereospecifically, to *E*-1,3-phenyl-1-butene. Despite the optical activity of the precatalysts no asymmetric induction was observed, and the product was obtained as a racemic mixture. It was noted that 1,4-benzoquinone plays a fundamental role for the catalytic reaction, being involved in catalyst activation. Further investigations are required to clarify these specific reactions.

## Supplementary Materials

Supplementary materials can be accessed at http://www.mdpi.com/1420-3049/16/2/1804/s1.

## References

[1] Espinet P., Soulantica K. (1999). Phosphine-pyridyl and related ligands in synthesis and catalysis. Coord. Chem. Rev..

[2] Maggini S. (2009). Classification of P,N-binucleating ligands for hetero- and homobimetallic complexes. Coord. Chem. Rev..

[3] Schoenleber M., Hilgraf R., Pfaltz A. (2008). Chiral Bis(N-arylamino)phosphine-oxazolines: Synthesis and Application in Asymmetric Catalysis. Adv. Synth. Catal..

[4] Mothes E., Sentets S., Luquin M.A., Mathieu M., Lugan N., Lavigne G. (2008). New insight into the reactivity of pyridine-functionalized phosphine complexes of ruthenium(II) with respect to olefin metathesis and transfer hydrogenation. Organometallics.

[5] Roseblade S.J., Pfaltz A. (2007). Iridium-Catalyzed Asymmetric Hydrogenation of Olefins. Acc. Chem. Res..

[6] Cipot J., McDonald R., Ferguson M.J., Schatte G., Stradiotto G. (2007). Cationic and formally zwitterionic rhodium(I) and iridium(I) derivatives of a P,N-substituted indene: A comparative synthetic, structural and catalytic investigation. Organometallics.

[7] Kimura M., Uozomi Y. (2007). Development of New P-Chiral Phosphorodiamidite Ligands Having a Pyrrolo[1,2-c]diazaphosphol-1-one Unit and Their Application to Regio- and Enantioselective Iridium-Catalyzed Allylic Etherification. J. Org. Chem..

[8] Arrayas R.G., Adrio J., Carrettero J.C. (2006). Recent applications of chiral ferrocene ligands in asymmetric catalysis. Angew. Chem. Int. Ed..

[9] Giuri P.J., Saunders C.P. (2004). The development of bidentate P,N ligands for asymmetric catalysis. Adv. Synth. Catal..

[10] Franciò G., Drommi D., Graiff C., Faraone F., Tiripicchio A. (2002). Synthesis of phosphonito,N and phosphito,N ligands based on quinolines and (R)-binaphthol or substituted biphenol and their rhodium(I), palladium(II) and platinum(II) complexes. Inorg. Chim. Acta.

[11] Brumel J.M., Legrand O., Reymond S., Buono G. (1999). First Iminodiazaphospholidines with a Stereogenic Phosphorous Center. Application to Asymmetric Copper-Catalyzed Cyclopropanation. J. Am. Chem. Soc..

[12] Bluhm M.E., Folli C., Walter O., Doering M. (2005). Nitrogen- and phosphorus-coordinated nickel(II) complexes as catalysts for the oligomerization of ethylene. J. Mol. Cat. A Chem..

[13] Cheng H.-P., Liu Y.-H., Peng S.-M., Liu S.-T. (2003). New bulky phosphino-pyridine ligands. Palladium and nickel complexes for the catalytic polymerization and oligomerization of ethylene. Organometallics.

[14] Sun W.-H., Li Z., Hu H., Wu B., Yang H., Zhu N., Leng X., Wang H. (2002). Synthesis and characterization of novel nickel(II) complexes bearing N,P ligands and their catalytic activity in ethylene oligomerization. New J. Chem..

[15] Daugulis O., Brookhart M. (2002). Polymerization of ethylene with cationic palladium and nickel catalysts containing bulky nonenolizable imine-phosphine ligands. Organometallics.

[16] Keim W., Killat S., Nobile C.F., Suranna G.P., Englert U., Wang R., Mecking S., Schroeder D.L. (2002). Synthesis, characterization and catalytic activity of Pd(II) and Ni(II) complexes with new cyclic alpha-diphenylphosphino-ketoimines. Crystal structure of 2,6-diisopropyl-N-(2-diphenylphosphino-cyclopropylidene)aniline and of 2,6-diisopropyl-N-(2-diphenylphosphino-cyclohexylidene)aniline. J. Organomet. Chem..

[17] Sirbu D., Consiglio G., Gischig S. (2006). Palladium and nickel complexes of (P,N)-ligands based on quinolines: Catalytic activity for polymerization and oligomerization. J. Organomet. Chem..

[18] Aeby A., Consiglio G. (1999). P^N versus N^N ligands for the palladium-catalyzed alternating copolymerization of styrene and carbon monoxide. Inorg. Chim. Acta.

[19] Gsponer A., Schmid T.M., Consiglio G. (2001). Ligand-dependent diastereoselectivity in the palladium-catalyzed copolymerization of styrene with carbon monoxide. Helv. Chim. Acta.

[20] Braunstein P., Fryzuk M.D., Le Dall M., Naud F., Rettig S.J., Speiser F. (2000). Synthesis and structure of Pd(II) complexes containing chelating (phosphinomethyl)oxazoline P,N-type ligands. copolymerisation of ethylene/CO. J. Chem. Soc., Dalton Trans..

[21] Agostinho M., Braunstein P. (2007). Palladium(II) complexes with the new P,N-type ligand (2-oxazoline-2-ylmethyl)di-isopropylphosphine as intermediates in CO/ethylene or CO/methyl acrylate insertion. C.R. Chim..

[22] Agostinho M., Braunstein P., Welter R. (2007). Phosphinito- and phosphonito-oxazoline Pd(ii) complexes as CO/ethylene insertion intermediates: Synthesis and structural characterization. Dalton Trans..

[23] Agostinho M., Braunstein P. (2007). Structurally characterized intermediates in the stepwise insertion of CO-ethylene or CO-methyl acrylate into the metal-carbon bond of Pd(II) complexes stabilized by (phosphinomethyl)oxazoline ligands. Chem. Commun..

[24] Buono G., Toselli N., Martin D., Börner A. (2008). Chiral Diazaphospholidine Ligands in Phosphorus Ligands in Asymmetric Catalysis.

[25] Breeden S., Cole-Hamilton D.J., Foster D.F., Schwarz G.J., Will M. (2000). Rhodium-mediated asymmetric hydroformylation with a novel bis(diazaphospholidine) ligand. Angew. Chem. Int. Ed..

[26] Myagmarsuren G., Tkack V.S., Shmidt F.K., Mohamad M., Suslov D.S. (2005). Selective dimerization of styrene ot 1,3-diphenyl-1-butene with bis(beta-diketonato)palladium/boron trifloride etherate catalyst system. J. Mol. Cat. A Chem..

[27] Peng J., Li J., Qiu H., Jiang J., Jiang K., Mao J., Lai G. (2006). Dimerization of styrene to 1,3-diphenyl-1-butene catalyzed by palladium-Lewis acid in ionic liquid. J. Mol. Cat. A Chem..

[28] Lee D.W., Yi C.S. (2010). Chain-Selective and Regioselective Ethylene and Styrene Dimerization Reactions Catalyzed by a Well-Defined Cationic Ruthenium Hydride Complex: New Insights on the Styrene Dimerization Mechanism. Organometallics.

[29] Cabrero-Antonino J.R., Leyva-Perez A., Corma A. (2010). Iron-Catalysed Regio- and Stereoselective Head-to-Tail Dimerisation of Styrenes. Adv. Synth. Catal..

[30] Vogt D. (2010). Cobalt-Catalyzed Asymmetric Hydrovinylation. Angew. Chem. Int. Ed..

[31] RajanBabu T.V. (2003). Asymmetric Hydrovinylation Reaction. Chem. Rev..

[32] Pettit G.R., Singh S.B., Herald D.L., Lloyd-Williams P., Kantoci D., Burkett D.D., Barkoczy J., Hogan F., Wardlaw T.R. (1994). The Dolastatins. 17. Synthesis of Dolaproine and Related Diasteroisomers. J. Org. Chem..

[33] Xu X.-Y., Tang Z., Wang Y.-Z., Luo S.-W., Cun L.-F., Gong L.-Z. (2007). Asymmetric Organocatalytic Direct Aldol Reactions of Ketones with alpha-Keto acids and Their Application to the Synthesis of 2-Hydroxy-gamma-butyrolactones. J. Org. Chem..

[34] Luo S., Xu H., Li J., Zhang L., Mi X., Zheng X., Cheng J.-P. (2007). Facile evolution of asymmetric organocatalysts in water assisted by surfactant Brønsted acids. Tetrahedron.

[35] Bartoli G., Bosco M., Dalpozzo R., Giuliani A., Marcantoni E., Mercozzi T., Sambri L., Torregiani E. (2002). An Efficient Procedure for the Preparation of (E)-alpha-Alkylidene Cycloalkanones Mediated by CeCl_3_*7H_2_O-NaI System. Novel Methodology for the Synthesis of (S)-(-)-Pulegone. J. Org. Chem..

[36] Torisawa Y., Hashimoto A., Okouchi M., Limori T., Nagasawa M., Hino T., Nakagawa M. (1996). Manzamine C Congeners with Modified Azacyclic Rings: Synthesis and Biological Evaluation. Bioorg. Med. Chem. Lett..

[37] Legrand O., Brumel J.M., Constantieux T., Buono G. (1998). Totally Stereoselective P-O to P-C Migration Rearrangement: Application to the Synthesis of New Chiral o-Hydroxyaryl Phosphine Oxides. Chem. Eur. J..

[38] del Campo O., Carbayo A., Cuevas J.V., García-Herbosa G., Muñoz A. (2009). Isomeric Preference in Complexes of Palladium(II) with Chelating P,N-Donor Ligands. Eur. J. Inorg. Chem..

[39] Morita D.K., Stille J.K., Norton J.R. (1995). Methyl/Phenyl Exchange between Palladium and a Phosphine Ligand. Consequences for Catalytic Coupling Reactions. J. Am. Chem. Soc..

[40] D'Amora A., Fanfoni L., Cozzula D., Guidolin N., Zangrando E., Felluga F., Gladiali S., Benedetti F., Milani B. (2010). Addressing the Poly- to Oligo-ketone selectivity in styrene Carbonylation Catalyzed by Palladium/bpy Complexes. Effect of the 6-Alkyl Substitution. Organometallics.

[41] Durand J., Scarel A., Milani B., Seraglia R., Gladiali S., Carfagna C., Binotti B. (2006). Palladium-Promoted Carbon Monoxide/Ethylene/Styrene Terpolymerization Reaction: Throwing Light on the Different Reactivity of the Two Alkenes. Helv. Chim. Acta.

[42] Drent E., Budzelaar P.H.M. (1996). Palladium catalyzed alternating copolymerization of alkenes and carbon monoxide. Chem. Rev..

[43] Milani B., Anzilutti A., Vicentini L., Sessanta o Santi A., Zangrando E., Geremia S., Mestroni G. (1997). Bis-chelated palladium(II) complexes with nitrogen-donor chelating ligands are efficient catalyst precursors for the CO/styrene copolymerization reaction. Organometallics.

[44] Scarel A., Durand J., Franchi D., Zangrando E., Mestroni G., Carfagna C., Mosca L., Seraglia R., Consiglio G., Milani B. (2005). Mono- and dinuclear bioxazoline-palladium complexes for the stereocontrolled synthesis of CO/styrene polyketones. Chem. Eur. J..

[45] Axet M.R., Amoroso F., Bottari G., D'Amora A., Zangrando E., Faraone F., Drommi D., Saporita M., Carfagna C., Natanti P., Seraglia R., Milani B. (2009). Application of chiral amine-imine ligands in palladium-catalyzed polyketones synthesis: Effect of ligand backbone on the polymer stereochemistry. Organometallics.

