# Supplementary material for: New Chiral P-N Ligands for the Regio- and Stereoselective Pd-Catalyzed Dimerization of Styrene
Source: Molecules. 2011 Feb 22;16(2):1804–24. doi: 10.3390/molecules16021804 (PMC6259647; doi:10.3390/molecules16021804)
Supplement: Supplementary file 1 [file molecules-16-01804-s001.pdf]

Supplementary Materials

Figure 1S. Homonuclear COSY spectrum of **1c**, in CD_2Cl_2 at 238 K (the *ortho* and *meta* protons of 4-picoline are evidenced).

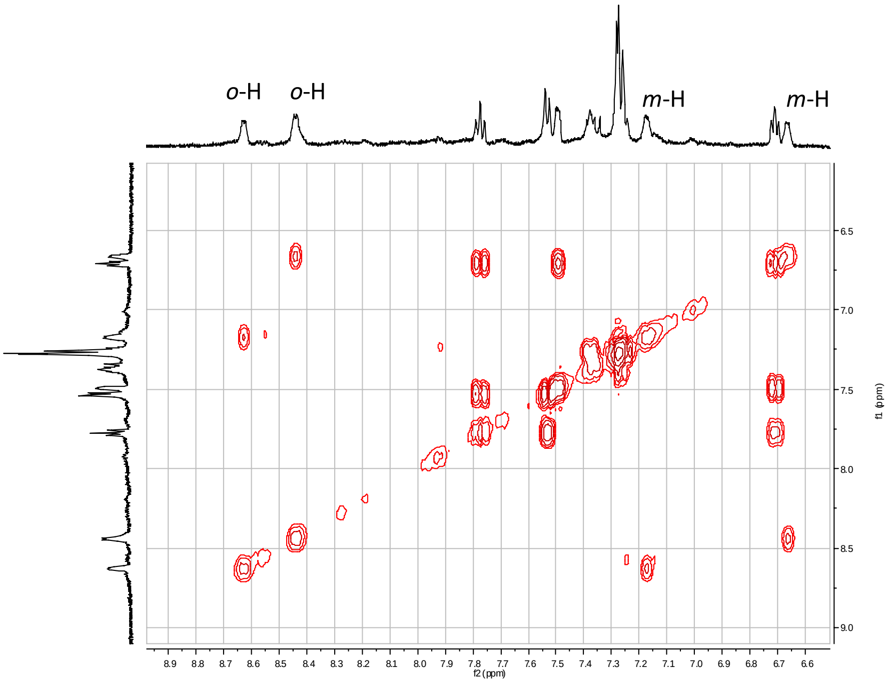

Figure 2S. NMR spectra of **1b**, in CD_2Cl_2: variation with temperature (the *ortho, para* and *meta* protons of pyridine are evidenced).

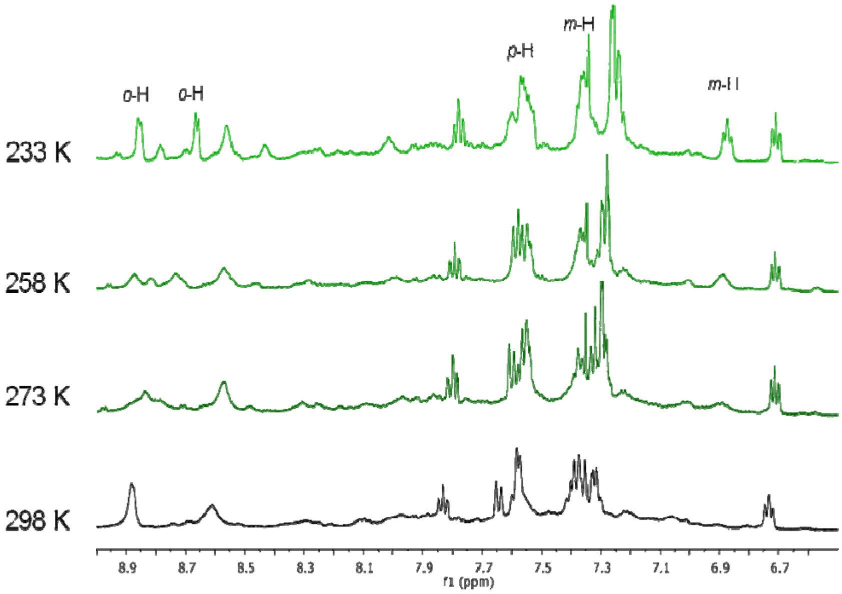

Figure 3S. Homonuclear COSY spectrum of **1b**, in CD_2Cl_2 at 233 K (the *ortho, para* and *meta* protons of pyridine are evidenced).

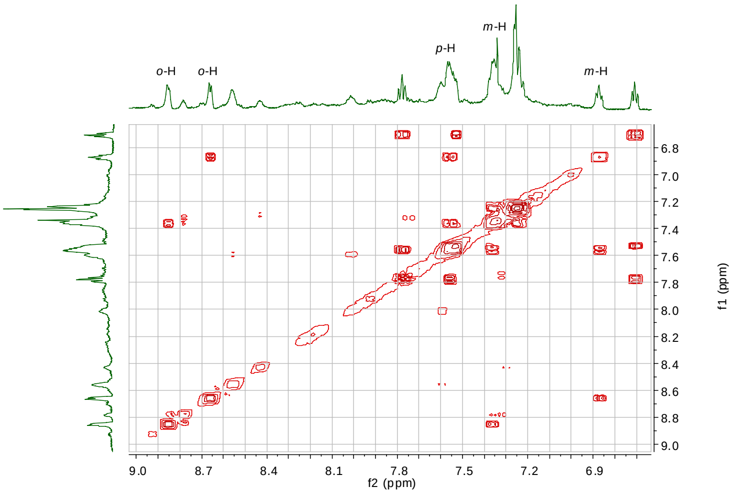

Figure 4S. NMR spectra of **1d**, in CD_2Cl_2: variation with temperature (the *ortho* and *meta* protons of 4-CF_3-pyridine are evidenced).

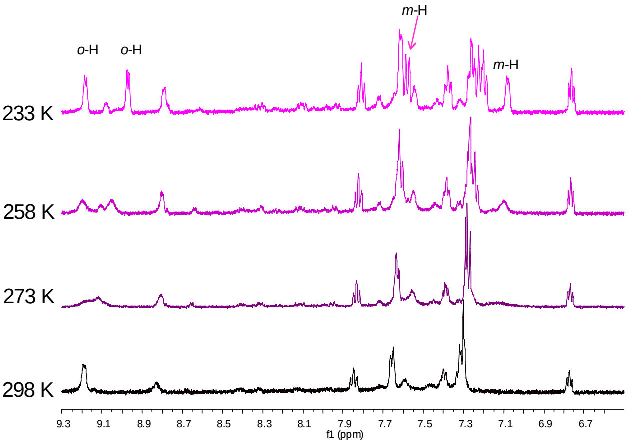

Figure 5S. Homonuclear COSY spectrum of **1d**, in CD_2Cl_2 at 238 K (the *ortho* and *meta* protons of 4-CF_3-pyridine are evidenced).

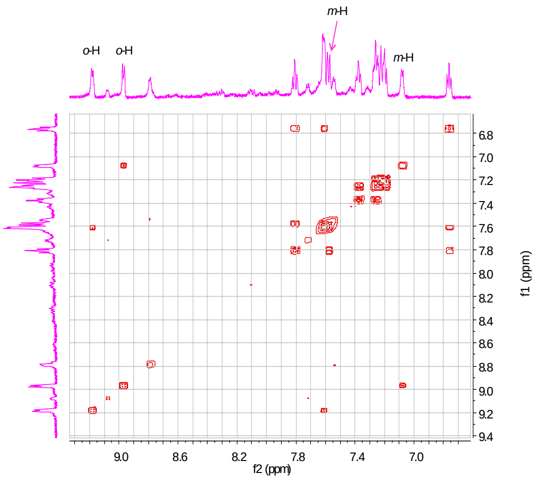

Figure 6S. CD spectra of complexes **1b-d** (1×10^{-4} M in CH_3OH).

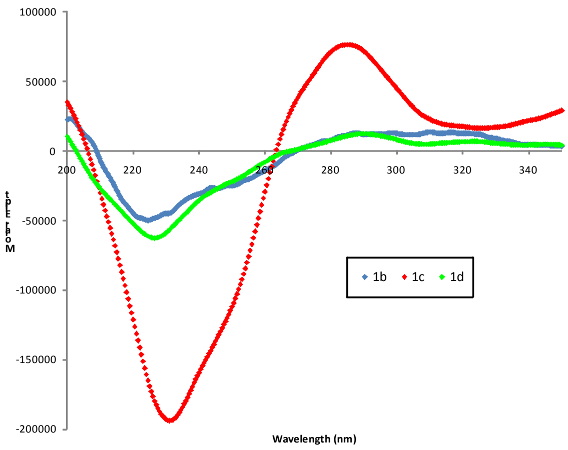

Figure 7S. ^{31}P-{^{1}H} NMR spectra of **2b**, in CD_2Cl_2: variation with temperature.

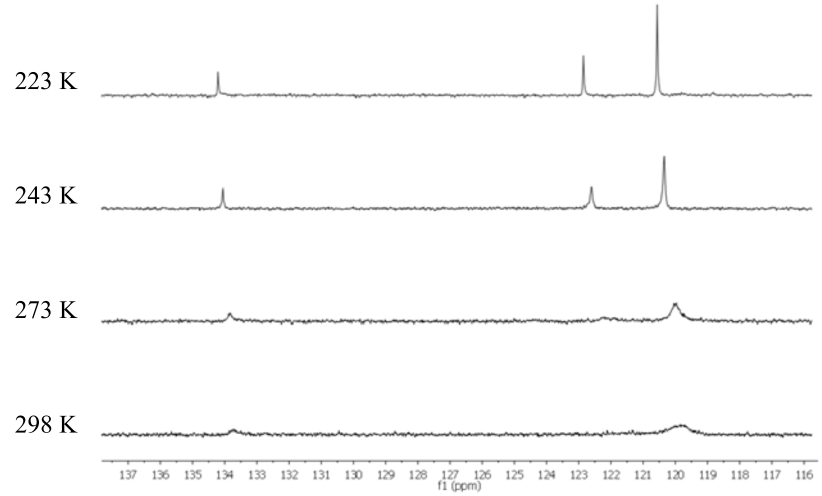

Figure 8S. ^{1}H NMR spectra of **2b**, in CD_2Cl_2 (aromatic region): variation with temperature (the *ortho* and *meta* protons of pyridine ring of P-N ligand are evidenced).

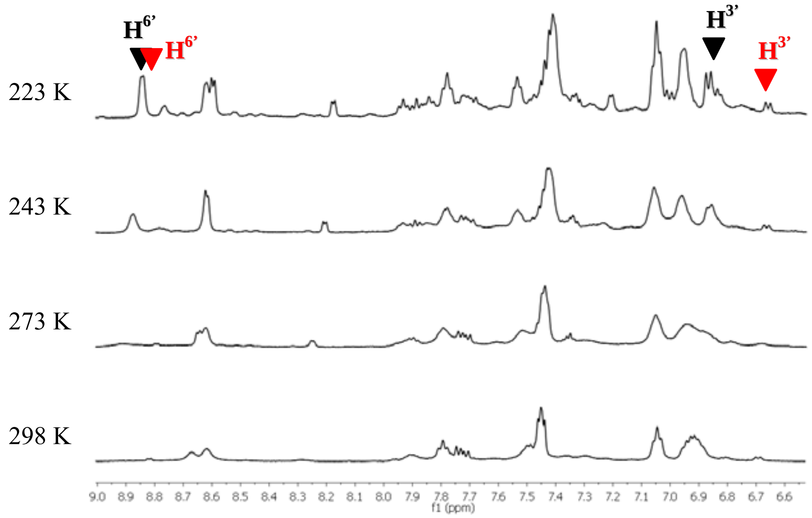

Figure 9S. ^{1}H NMR spectra of **2b**, in CD_2Cl_2 (portion of aliphatic region): variation with temperature ($Pd\text{-}CH_3$ groups are evidenced).

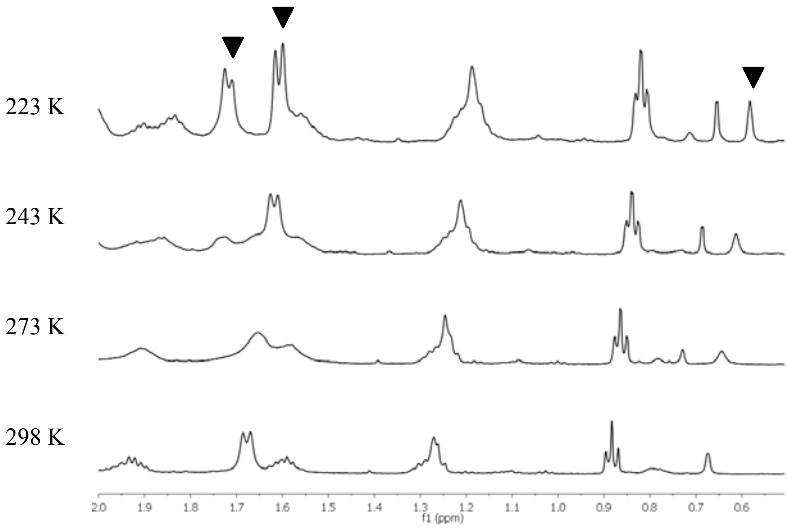

Figure 10S. $^{1}H,^{31}P$-HMBC NMR spectra of **2b**, in CD_2Cl_2 at 223 K.

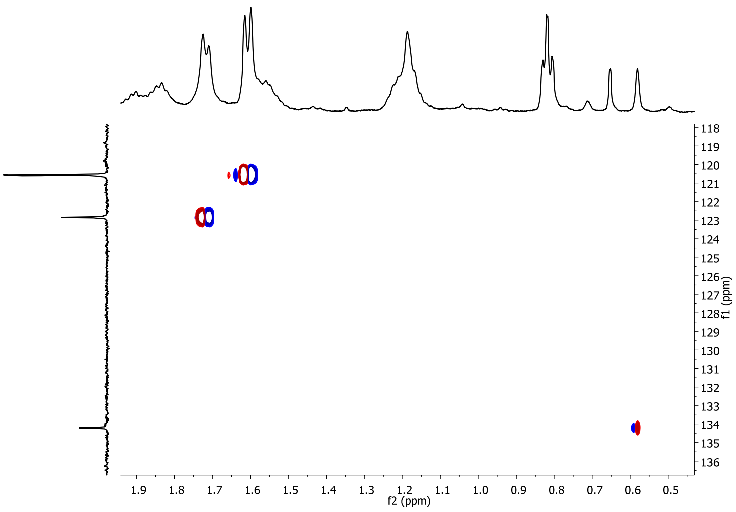

Table 1S. Selected coordination bond distances (Å) for **1c**.

Pd(1)-N(1)	2.033(15)
Pd(1)-N(2)	2.131(13)
Pd(1)-N(7)	2.09(3)
Pd(1)-P(2)	2.224(10)
Pd(2)-N(5)	2.075(16)
Pd(2)-N(6)	2.095(13)
Pd(2)-N(3)	2.05(2)
Pd(2)-P(1)	2.208(13)

Table 2S. Styrene dimerization: effect of temperature. Precatalyst: **1c**.[a]

Run	T (K)	TOF[b]	Conversion (%)[c]
1	303	9	8.81
2	323	67	16.70
3	343	159	24.83

[a] Reaction conditions: n_{Pd} = 1.27 × 10^{-5} mol, [BQ]/[Pd] = 40, [styrene]/[Pd] = 6800, styrene V = 10 mL, TFE V = 20 mL; [b] Turnover frequency = moles of product obtained per mole of Pd after 2 h; [c] Conversion determined by HRGC analysis after 24 h.

Figure 11S. Styrene dimerization: effect of [styrene]/[Pd]. Precatalyst: **1c**.

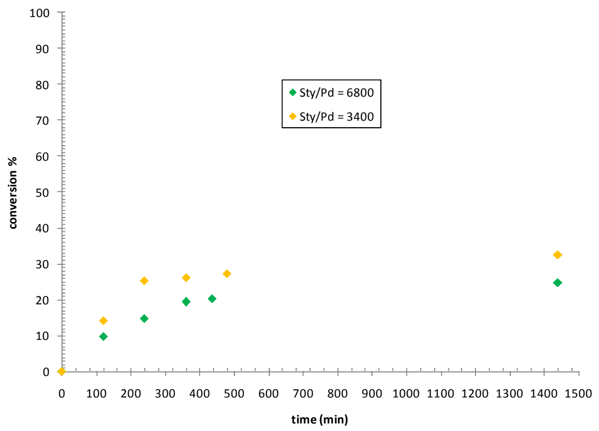

Reaction conditions: see Table 2S, n_{Pd} =1.27 × 10^{-5} mol for [styrene]/[Pd] = 6800, n_{Pd} = 2.54 × 10^{-5} mol for [styrene]/[Pd] = 3400, T = 343 K.

Table 3S. Styrene dimerization: effect of [BQ]/[Pd]. Precatalyst: **1b**.[a]

Run	[BQ]/[Pd]	TOF[b]	Conversion (%)[c]
1	0	27	12.24
2	20	190	39.11
3	40	209	48.87
4	80	345	81.88

[a] Reaction conditions: n_{Pd} = 2.54 × 10^{-5} mol, [styrene]/[Pd] = 3400, styrene V = 10 mL, TFE V = 20 mL; [b] Turnover frequency = moles of product obtained per mole of Pd after 2 h; [c] Conversion determined by HRGC analysis after 24 h.
